# Sexual and reproductive health service utilization among patients with podoconiosis in Wolaita Zone, South Ethiopia: a multilevel mixed-effect analysis

**DOI:** 10.3389/frph.2025.1562495

**Published:** 2025-08-18

**Authors:** Temesgen Lera Abiso, Amene Abebe Kerbo, Eskinder Wolka Woticha

**Affiliations:** School of Public Health, College of Medicine and Health Sciences, Wolaita Sodo University, Wolaita, Ethiopia

**Keywords:** sexual and reproductive health, sexual and reproductive health service utilization, podoconiosis, Wolaita Zone, sexual and reproductive health services

## Abstract

**Background:**

Sexual and reproductive health (SRH) services are essential for promoting the wellbeing of individuals and communities. Achieving universal health coverage is unattainable without ensuring equitable access to SRH services and upholding sexual and reproductive rights. Podoconiosis, a neglected tropical disease and non-filarial form of elephantiasis, is caused by prolonged barefoot exposure to irritant volcanic soils. Although SRH services have been increasingly integrated into primary healthcare systems in Ethiopia, people affected by podoconiosis continue to face substantial barriers in accessing these services due to stigma, mobility limitations, and socioeconomic constraints. In this context, the present study aims to assess the utilization of SRH services and the factors associated with their utilization among patients with podoconiosis in Wolaita Zone, South Ethiopia.

**Methods:**

A community-based cross-sectional study was conducted among 836 patients with podoconiosis in Wolaita Zone from 12 November to 20 December 2024. Multistage sampling was used to select participants, and data were collected through face-to-face interviews. A multilevel logistic regression analysis was performed to identify the factors associated with SRH service utilization. Statistical significance was set at *p* < 0.05, and adjusted odds ratios (AOR) with 95% confidence intervals (CI) were reported.

**Results:**

The study found that 154 respondents (18.4%; 95% CI: 16%, 21%) utilized SRH services within the past 12 months. The factors significantly associated with SRH service utilization included the following: age groups 18–29 years (AOR = 3.57; 95% CI: 1.55–8.25), 30–34 years (AOR = 2.89; 95% CI: 1.30–6.40), and 35–39 years (AOR = 5.06; 95% CI: 2.45–10.45); prior experience with health services (AOR = 2.39; 95% CI: 1.56–4.93); family awareness of podoconiosis etiology (AOR = 3.60; 95% CI: 2.13–6.21); positive attitude toward SRH services (AOR = 5.80; 95% CI: 3.16–10.70); family support (AOR = 2.47; 95% CI: 1.47–4.14); and autonomy in using household financial resources for healthcare (AOR = 2.05; 95% CI: 1.18–3.57).

**Conclusion:**

The utilization of SRH services among patients with podoconiosis was found to be low. The significant factors associated with SRH service utilization included the age of respondents, prior experience with health services, family awareness of podoconiosis etiology, family support, autonomy in using household financial resources for healthcare, and positive attitudes toward SRH services.

## Introduction

The World Health Organization (WHO) defines reproductive health as a state of complete physical, mental, and social wellbeing and not merely the absence of disease or infirmity in all matters relating to the reproductive system and its functions and processes. This definition implies that individuals have the ability to enjoy a satisfying and safe sexual life, as well as the freedom to decide if, when, and how often to reproduce (1).

People need access to accurate information and safe, effective, affordable, and acceptable contraceptive methods of their choice to maintain their sexual and reproductive health (SRH). In addition, they must be empowered and informed to protect themselves against sexually transmitted infections (STIs) (2).

Sustainable Development Goals (SDGs) (3.7) state that ensuring universal access to sexual and reproductive healthcare services, including to family planning (FP) and information and education, and the integration of reproductive health into national strategies and programs, is critical for nations to achieve this goal (3). Universal health coverage, which involves enhancing health service use and guaranteeing equity (4), requires the realization of universal access to sexual and reproductive health and rights, which is the cornerstone of SDGs (3).

Although there is global consensus on the importance of ensuring equity in health service utilization for all populations, individuals affected by neglected tropical diseases (NTDs) remain among the most marginalized groups in accessing healthcare. Podoconiosis, a non-infectious form of lymphoedema, results from prolonged barefoot exposure to irritant volcanic soils. It is estimated to affect approximately 4 million people worldwide, primarily in highland tropical and subtropical regions across 17 countries in Africa, Central and South America, and South and Southeast Asia (5).

Ethiopia shares the highest global burden of podoconiosis; over 35 million people are at risk, and more than 1.5 million live with the disease across 345 districts in the country (6). In endemic areas, the rate of prevalence ranges from 5% to 10% (7, 8). Podoconiosis is strongly associated with stigma from community members and health professionals, which contributes to self-stigma and reduced self-confidence among affected individuals. This stigma discourages affected persons from seeking healthcare and results in poor medical care (9–11). The stigma related to podoconiosis profoundly impacts reproductive health outcomes, leading to poor marriage prospects, reluctance to marry affected individuals or their family members, discrimination in marital relationships, and suboptimal medical care (9, 10, 12–16).

Patients affected by podoconiosis may adopt either positive or negative coping mechanisms in response to the stigma associated with the disease. Negative coping behaviors that adversely affect reproductive health include avoiding marriage to non-affected individuals, engaging in premarital sex, divorce, and avoiding treatment (13, 16).

Despite the substantial impact of podoconiosis on reproductive health, there is a lack of data on SRH service utilization among affected patients in Ethiopia, including the study area. Therefore, this study aims to assess SRH service utilization and its associated factors among patients with podoconiosis in Wolaita Zone, South Ethiopia.

### Theoretical framework of the study

Research demonstrates that theory-driven investigations play a vital role in forecasting human behaviors, particularly in the context of utilizing sexual and reproductive health services, including Human Immune Virus (HIV) testing (17).

The health and medical fields typically employ the Andersen theoretical models and health belief model to elucidate the utilization of services (18–20). The Andersen model is the more widely used theoretical model for analyzing the predictors of health service utilization among the two. The Andersen model for health service utilization provides a framework that permits a systematic identification of factors that influence individual decisions to use (or not use) available healthcare services (21, 22). When describing the need for research due to a dearth of prior studies on the utilization of SRH services among patients with podoconiosis, this model is appropriate.

This study is grounded in the health care utilization model initially proposed by Anderson and Newman (22). Over time, this model has undergone several revisions to address barriers that hinder healthcare access for vulnerable populations (23). The model comprises three primary components: predisposing factors, enabling factors, and need-for-care factors, all of which can either facilitate or obstruct the use of health services by individuals (24). Predisposing factors encompass demographic traits, social structural elements, and an individual's fundamental beliefs, attitudes, and knowledge regarding health services (24). Enabling factors pertain to the resources available, whether at the individual or community level. Need factors refer to the illnesses, conditions, and health statuses that necessitate health services (24). This model has found applications across various disciplines, including sociology, medicine, public health, and psychology. It has been particularly useful in analyzing the utilization of healthcare services, such as HIV testing among young women in Trinidad and Tobago (25).

## Methods and materials

### Study setting and design

**Figure 1:**
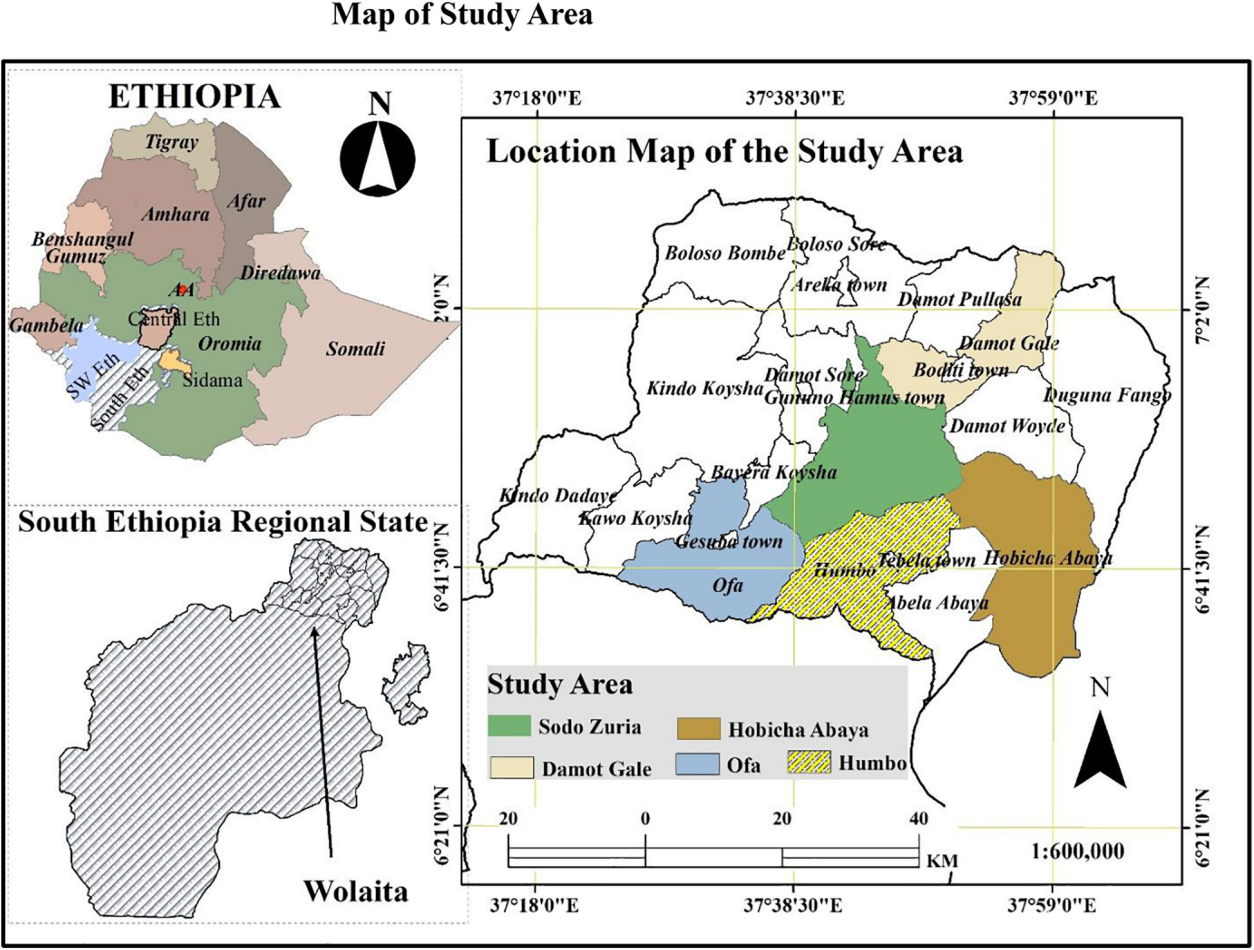
Maps illustrating Ethiopia, the South Ethiopia Region, Wolaita Zone, and the study districts were developed using shapefiles obtained from the Humanitarian Data Exchange website (https://data.humdata.org/dataset/cod-ab-eth). These shapefiles are in the public domain and freely available for use.

The study was conducted in Wolaita Zone, one of the 12 administrative zones in the South Ethiopia Region. Wolaita Zone is located approximately 324km south of Addis Ababa, the capital city of Ethiopia. It consists of 22 districts and 7 town administrations (Figure 1) and is recognized as one of the podoconiosis-prevalent zones in the country. Geographically, Wolaita Zone is bordered to the south by Gamo and Gofa Zones, to the west by the Omo River (separating it from Dawuro), to the northwest by Kambata Tembaro, to the north by Hadiya, to the northeast by the Oromia Region, to the southeast by Lake Abaya, and to the east by the Bilate River (separating it from the Sidama Region). The administrative center of the zone is Wolaita Sodo city. Covering an area of approximately 451,170.7 ha, Wolaita Zone has a total population of 2,857,461, of whom 1,402,069 are male and 1,455,392 are female. The zone comprises 414,192 households, with an average household size of approximately five persons across 369 kebeles (290 rural and 79 urban). Health infrastructure includes 362 functional health posts, 69 health centers, and 12 hospitals (8 governmental primary hospitals, 2 non-governmental organization (NGO) primary hospitals, 1 NGO general hospital, and 1 comprehensive specialized hospital). According to estimates, there are 14,888 podoconiosis cases in the zone (26).

### Source and study population

The source population for this study included all reproductive-aged women (15–49 years) and men (15–64 years) with podoconiosis residing in Wolaita Zone. Podoconiosis cases were confirmed through clinical or laboratory methods at health facilities and recorded as patients with podoconiosis within the districts of Wolaita Zone. Those podoconiosis patients who met the study's inclusion criteria constituted the study population.

### Inclusion and exclusion criteria

#### Inclusion criteria

This study included reproductive-aged women (15–49 years) and men (15–64 years) with podoconiosis residing in Wolaita Zone, South Ethiopia. Participants were patients with podoconiosis within these age groups who were sexually active and willing to participate in the study.

#### Exclusion criteria

Individuals with podoconiosis who were severely ill during data collection, unable to communicate, or had mental health conditions preventing participation were excluded from the study. In addition, patients experiencing acute pain related to podoconiosis at the time of data collection were excluded.

### Sample size and sampling procedure

#### Sample size determination

The sample size for these objectives was calculated based on the single population proportion formula with the assumption of 50% utilization of reproductive health service among patients with podoconiosis in Ethiopia, since there is no existing study on people living with podoconiosis; the margin of error was 5% with 95% CI, a 10% non-response rate, and a design effect of 2

$$n=\frac{{\left[{\left(\;Z_{1-\alpha /2}\;\right)}^{2}\times p(1-p)\;\right]}_{\mathrm{Def}}}{d^{2}}$$

Z = Standard normal distribution value at 95% CI = (1.96)^2^. where —Z is the standard normal distribution value at 95% CI = (1.96)^2^.

*d* = 0.05(5% margin of error).

*P* = 0.5 (50%).

Therefore, the sample size required

$n=\frac{\left[{\left(1.96\;\right)}^{2}\times 0.5(0.5)\right]}{0.05^{2}}2\;=768$ by adding 10% non-response rate, and the required sample size calculated was 845.

#### Sampling procedure

**Figure 2:**
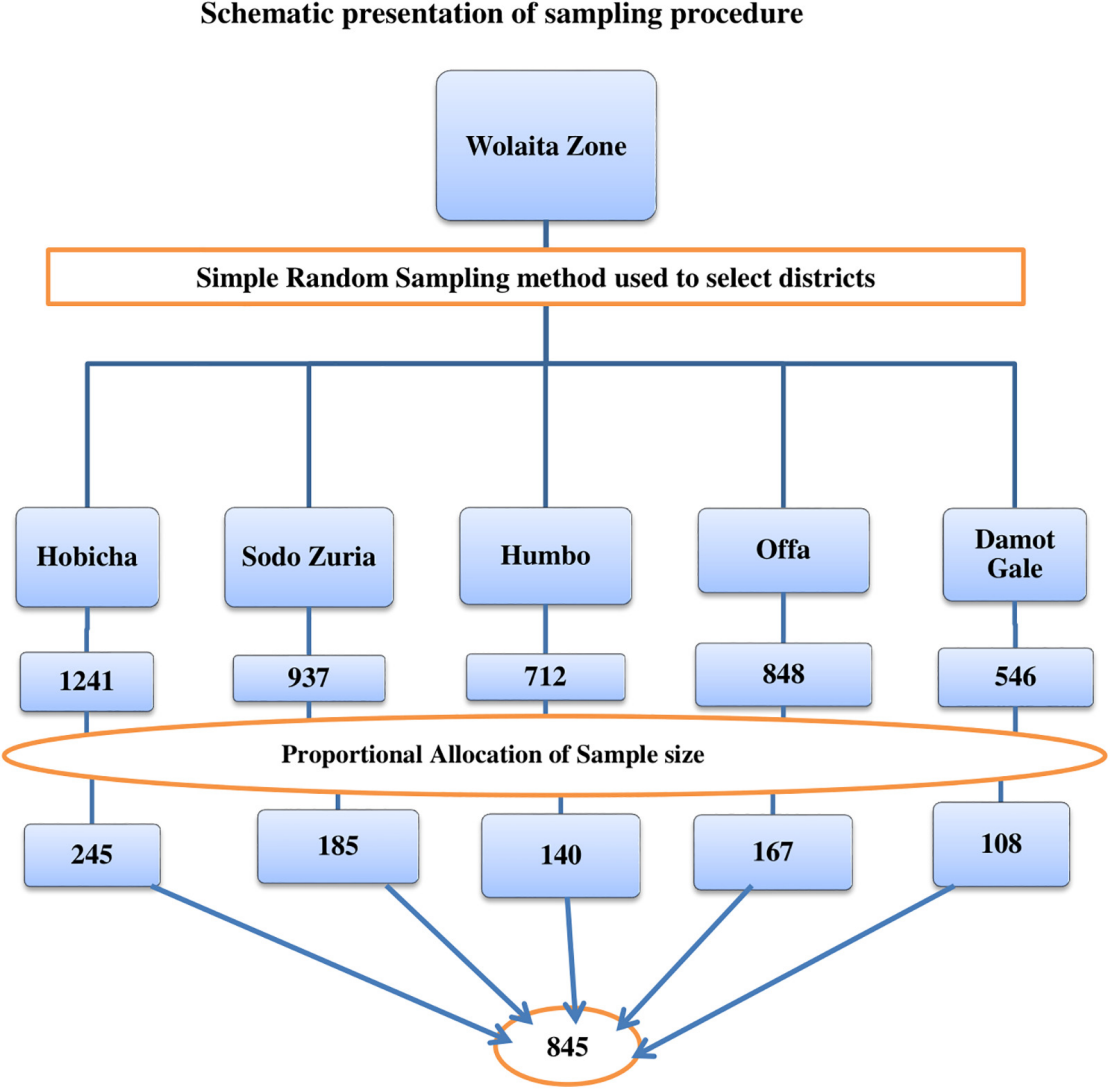
A diagrammatic representation of the sampling technique for a study on SRH utilization and associated factors among patients with podoconiosis in Woliata Zone, South Ethiopia, 2024.

From the 22 districts and 7 town administrations in Wolaita Zone, five districts were purposively selected for this study: Damot Gale, Sodo Zuria, Humbo, Hobicha, and Offa. The total number of reproductive-age adults with podoconiosis in these districts was obtained from the district NTD focal persons. Within the selected districts, kebeles with eligible podoconiosis patients were identified, and the calculated total sample size was proportionally allocated to each district (Figure 2). Data collection was carried out at the household level in collaboration with health extension workers from the selected kebeles. Podoconiosis patients were registered at both the district health office and the health post levels, with NTD focal persons and health extension workers having a detailed knowledge of these patients. A computer-generated simple random sampling technique was used to select study participants from the registries. In households with more than one eligible podoconiosis patient, one participant was chosen using a lottery method. Health extension workers assisted data collectors and supervisors by guiding them to the homes of selected patients.

#### Data collection procedure

Data were collected using an interviewer-administered semistructured questionnaire adapted from relevant literature. The Kobocollect apk on smartphones was employed for data collection. Trained data collectors conducted face-to-face interviews under the supervision of the study team and the principal investigator. Participants were selected through simple random sampling using a house-to-house approach, guided by kebele volunteers and health extension workers. Five Master of Public Health professionals with prior experience in health-related data collection served as data collectors, while two additional Master of Public Health professionals supervised the data collection process.

### Variables of the study

#### Dependent Variable

Sexual and reproductive health service utilization.

#### Independent variables

**Predisposing factors**: Sociodemographic variables such as age, sex, residence, marital status, education, partner education, occupation, lack of awareness, and difficulty to move.

**Enabling factors**: Wealth index, partner occupation, involvement in income-generating activities, involvement in community activities, decision-making ability, use of shoes, regular washing of feet with soap and water, awareness of family members about the cause of disease, family support, family member with the disease, experience of health service use, accessibility of services, availability of services, acceptability of services, quality of services, health professional approach, presence of podoconiosis prevention team at health facility/community, presence of organizations supporting patients, follow-up of patients, presence of trained health personnel, and presence of networking.

**Need-for-care factors:** Presence of other disease, years lived with the disease, frequent acute attacks, complicated disease status, and stigma.

### Data quality management

To ensure data quality, several measures were implemented throughout the study. First, a pretest was conducted among 5% of the total sample size in Damot Sore, a district not included in the actual study area. Based on the pretest findings, necessary revisions were made to the data collection tool. In addition, data collectors and supervisors were carefully recruited and provided with comprehensive training to ensure that they fully understood the study procedures and their respective roles. Data collection was carried out using the Kobocollect apk. Supervisors closely monitored and evaluated the data collection process daily, reporting any issues encountered in the field. The principal investigator coordinated and oversaw the entire data collection process, ensuring adherence to protocols and addressing any challenges promptly. Daily debriefing sessions were held each morning with data collectors, supervisors, and the principal investigator to review progress and resolve emerging issues. At the end of each day, collected data were checked for completeness, consistency with the data collection tool, and potential errors. Data were then uploaded to the server and reviewed for accuracy and time spent per interview. Following data collection, the dataset was exported to STATA version 14 for further exploration. At this stage, missing values and other inconsistencies were identified and corrected to ensure high-quality data for analysis.

### Operational definitions and measurement

**Sexual and reproductive health service utilization**: It is the use of one of the comprehensive sexual and reproductive health services such as FP, Antenatal care (ANC), delivery service, postnatal care (PNC), postabortion service (PAC), HIV testing and counseling service, STIs, and HIV treatment service (27).

**SRH care service utilization**: SRH service utilization was defined as the use of at least one of the following services within the past 12 months: FP, ANC, delivery care, PNC, PAC, HIV testing and counseling, or treatment for STIs and HIV. For male participants, only HIV testing and counseling services and STI/HIV treatment services were assessed.

Patients with podoconiosis were asked whether they had utilized any of these SRH services during the preceding 12 months. Responses were recorded on a binary scale, with “Yes” indicating the utilization of at least one SRH service and “No” indicating no utilization. To confirm a positive (“Yes”) response, participants were further asked to specify which SRH services they had accessed*.*

**Accessibility**: This refers to non-discrimination to access, physical accessibility of health facility, economic accessibility (affordability), and information accessibility (28).

**Attitude toward SRH**: Attitude toward SRH services was assessed using eight items measured on a five-point Likert scale, ranging from 1 (strongly disagree) to 5 (strongly agree). The overall attitude of participants was classified as positive if their total score was above the mean and negative if their score was below the mean. The internal consistency of the attitude scale was high, with a Cronbach's alpha coefficient of 0.884.

**Wealth index**: Principal component analysis was conducted to generate a composite variable for household wealth status. The assessment of household asset ownership was based on tools adapted from the Ethiopian Demographic and Health Survey, considering both rural and urban contexts. Wealth scores were calculated separately for rural and urban households to account for contextual differences in asset ownership. The two scores were then merged, and households were categorized into three wealth groups: poor, middle, and rich.

### Data analysis procedures

The collected data were exported from Kobocollect apk to STATA version 14 for cleaning, recoding, and categorization. Descriptive statistics, including mean, standard deviation, and percentages, were used to summarize the data. Bivariable logistic regression was performed to identify candidate variables for multivariable analysis, with variables having a *p*-value < 0.25 included in the multilevel logistic regression model. A two-level regression analysis was conducted to identify the best-fitting model for predicting the outcome variable.

Multicollinearity was assessed using the variance inflation factor (VIF), and no variable had a VIF greater than 10. Model fitness was evaluated using the Akaike information criterion (AIC) and Bayesian information criterion (BIC). AORs with 95% confidence intervals (CI) were reported, and statistical significance was declared at a *p*-value < 0.05.

### Model building

To assess the effects of both individual-level (Level I) and community-level (district; Level II) factors on SRH service utilization, a two-level multilevel logistic regression model was developed, with individual podoconiosis patients nested within their respective districts/communities. Initially, a bivariable two-level logistic regression was performed, and variables with a *p*-value ≤ 0.25 were selected as candidates for the multivariable analysis. Both bivariable and multivariable multilevel binary logistic regression analyses were conducted using the STATA syntax melogit. Four hierarchical models were fitted as follows:

**Model I (Null Model):** This model included only a random intercept to estimate the overall variability in SRH service utilization among patients with podoconiosis across districts. It was used to assess the need for multilevel modeling by calculating the intraclass correlation coefficient (ICC).

**Model II (Individual-Level Predisposing Factors):** This model examined the effects of individual-level predisposing factors on SRH utilization, including variables that were significant in the bivariable analysis.

**Model III (Enabling and Need Factors):** This model assessed the effects of enabling and need factors on SRH service utilization, including statistically significant variables identified in the bivariable multilevel analysis.

**Model IV (Full Model):** This model simultaneously incorporated predisposing, enabling, and need factors to examine their combined effects on SRH utilization.

### Model fitness statistics

The amended final model's goodness of fit was assessed using the log-likelihood ratio (LLR), BIC, and AIC in relation to the earlier models. The best fit model was determined to be the one with the highest LLR value and the lowest BIC and AIC values (29).

### Multicollinearity

Multicollinearity was examined using a pseudo linear regression analysis, and VIF was set at a cutoff of 10 (30). There was no indication of high collinearity between the independent variables, according to the results (mean VIF = 1.87, minimum VIF = 1.19, maximum VIF = 3.32).

## Results

### Sociodemographic and economic characteristics of patients with podoconiosis

Out of 845 eligible podoconiosis patients, 836 participated in the study, resulting in a response rate of 98.93%. Of these, 306 (36.6%) were male and 530 (63.4%) were female. In terms of age distribution, 401 participants (48.0%) were in the 35–44 year age group, while 185 (22.1%) were aged 25–34 years. The majority of participants, 454 (54.3%), had no formal education, and 639 (76.4%) were married (Table 1).

**Table 1:** Sociodemographic characteristics of patients with podoconiosis in Woliata Zone, South Ethiopia, 2024.

Sociodemographic characteristics	Description	Frequency (%)
Sex of the participant	Male	306 (36.6)
	Female	530 (63.4)
Age of the participants	18–29	117 (14)
	30–34	115 (13.7)
	35–39	226 (27)
	40–44	196 (23.5)
	≥45	182 (21.8)
Educational status of respondent	No formal education	454 (54.3)
	Primary education	297 (35.5)
	Secondary and above	85 (10.2)
Occupational status of the respondent	Farmer	755 (90.3)
	Merchant	51 (6.1)
	Government and private worker	30 (3.59)
Marital status of the respondent	Married	639 (76.4)
	Single	58 (6.9)
	Divorce	22 (2.6)
	Widowed	117 (14)
Educational status of the partner	No formal education	392 (46.9)
	Primary	181 (21.7)
	Secondary and above	66 (7.9)
Occupational status of the partner	Farmer	597 (93.4)
	Merchant	21 (3.3)
	Government and private worker	21 (3.3)
Wealth index	Poor	335 (40)
	Middle	336 (40.2)
	Rich	165 (19.7)
Residence	Rural	503 (60.2)
	Urban	333 (39.8)

### Disease-related characteristics among patients with podoconiosis

Of the 836 respondents included in this study, 194 (23.2%) had lived with podoconiosis for 1–5 years, while 266 (31.8%) had lived with the disease for 6–10 years. More than half of the respondents, 488 (57.5%), reported experiencing more than one acute attack in the past 12 months. In addition, 169 participants (20.2%) reported having at least one family member affected by the disease. The majority of families of participants, 482 (57.7%), were aware of the cause of podoconiosis (Table 2).

**Table 2:** Disease-related characteristics of patients with podoconiosis in Wolaita Zone, South Ethiopia, 2024.

Characteristics	Description	Frequency	Percent
Years lived with the disease	1–5 years	194	23.2
	6–10 years	266	31.8
	11–15	160	19.1
	16–20	117	14
	≥20	99	11.9
Acute attack more than 1 in a year	Yes	481	57.5
	No	355	42.5
Family members with the disease	Yes	169	20.2
	No	667	79.8
Fear of stigma	Yes	306	36.6
	No	530	63.4
Support from the family	Yes	408	48.8
	No	428	51.2
Support from the government	Yes	252	30.1
	No	584	69.9
Presence of any other supporting organization	Yes	206	24.6
	No	630	75.4
Involvement in community affairs	Yes	709	84.8
	No	127	15.2
Wash feet regularly with soap and water	Yes	739	88.4
	No	97	11.6
Use of foot shoes	Yes	756	90.4
	No	80	9.6
Able to move from place to place	Yes	690	82.5
	No	146	17.5
Able to go to the health facility whenever sick	Yes	758	90.7
	No	78	9.3
Awareness of the family that the disease is not transmitted	Yes	482	57.7
	No	354	42.3
Awareness of the community that the disease is not transmitted	Yes	467	55.9
	No	369	44.1
Presence of other disease	Yes	236	28.2
	No	600	71.8

### Utilization of SRH services among patients with podoconiosis in Wolaita Zone, South Ethiopia, 2024

**Figure 3:**
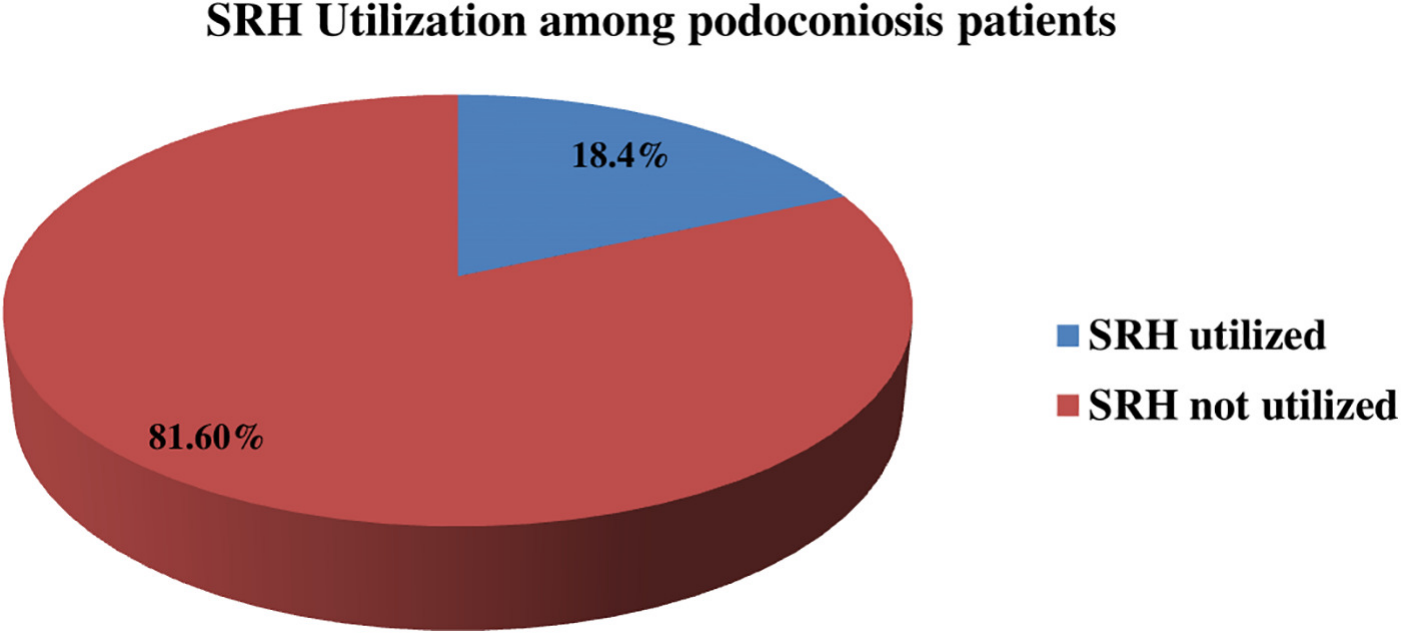
SRH utilization among patients with podoconiosis in Woliata Zone, South Ethiopia (*n* = 836).

**Figure 4:**
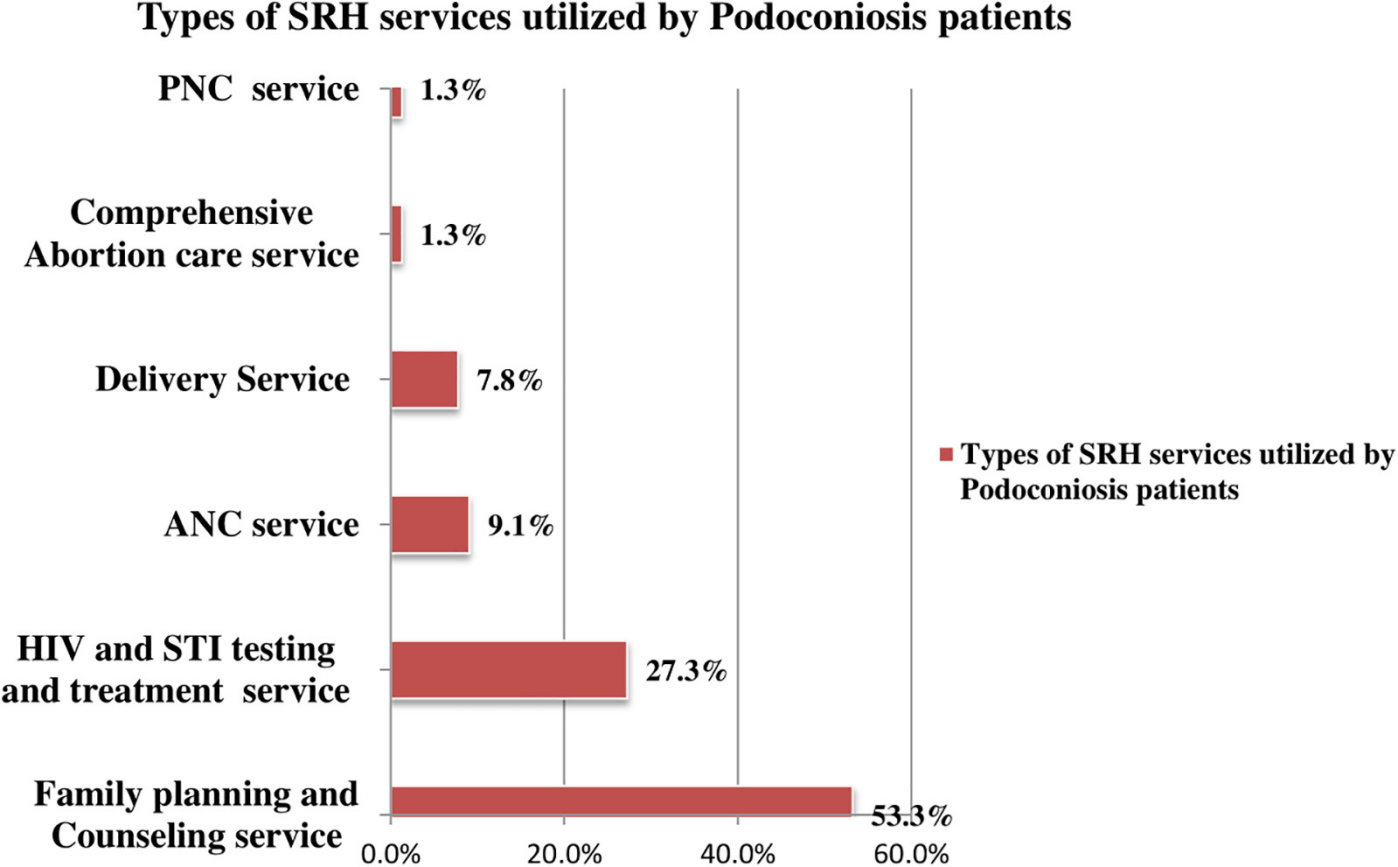
Types of SRH services by patients with podoconiosis in Woliata Zone, South Ethiopia, 2024 (*n* = 836).

Out of 836 study participants, 154 (18.4%; 95% CI: 16%–21%) reported utilizing SRH services within the past 12 months (Figure 3). Among these, the most commonly utilized services were FP and counseling (82; 53.3%), followed by HIV and STI testing and counseling (42; 27.3%), (ANC) services (14; 9.2%), and delivery care services (15; 7.8%) (Figure 4).

### SRH utilization status among male and female patients with podoconiosis

**Figure 5:**
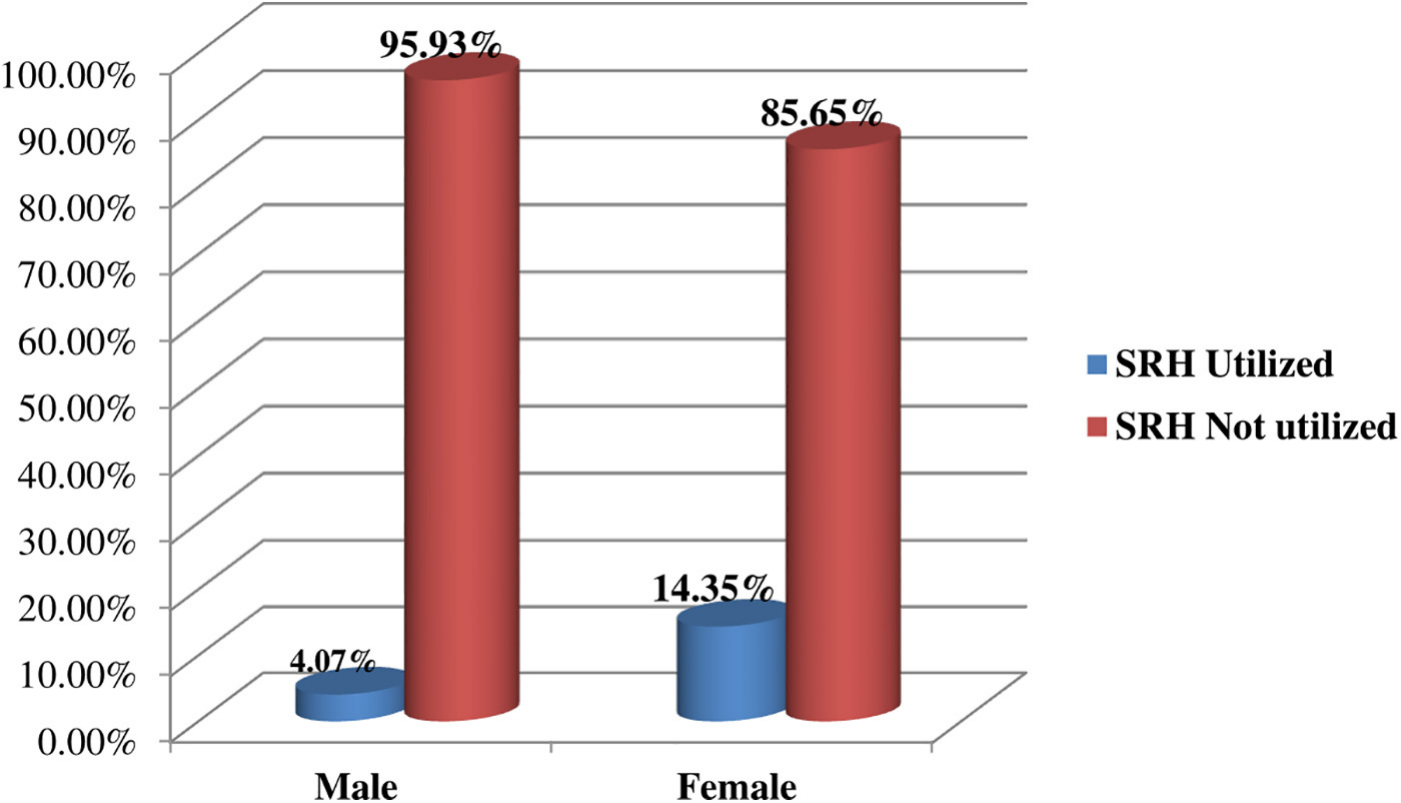
SRH utilization status among male and female patients with podoconiosis in Woliata Zone, South Ethiopia, 2024 (*n* = 836).

Of the patients who utilized SRH services, 120 (14.4%) were female and 34 (4.1%) were male. Female patients accessed a range of SRH services, including family planning, ANC, delivery care, PNC, comprehensive abortion care, and STI and HIV testing and counseling services. In contrast, male patients primarily utilized STI and HIV testing and counseling services (Figure 5).

### Multilevel mixed-effect analysis

#### Model statistics

To identify the predisposing, enabling, and need factors influencing SRH service utilization among female and male reproductive-age podoconiosis patients in Wolaita Zone, we performed a two-level mixed-effects multivariable logistic regression analysis. The random intercepts for SRH service utilization are presented in Table 3. In Model I (the empty model), the ICC indicated that 11% of the total variability in SRH utilization was attributable to differences between districts, while 89% was due to individual-level differences. The median odds ratio in the empty model was 2.11, suggesting significant variability in SRH service utilization across districts. Specifically, respondents from districts with higher SRH service utilization had a 2.11 times greater likelihood of utilizing SRH services compared with those from districts with lower utilization rates.

**Table 3:** Multilevel mixed-effect analysis of predisposing, enabling, and need factors associated with SRH service use among patients with podoconiosis in Wolaita Zone, South of Ethiopia 2024.

Characteristics	Categories	AOR 95% CI			
		Null model (I)	Model II	Model III	Model IV
Predisposing factors					
Age of respondent	18–29		3.34 (1.54, 6.7)		3.57 (1.56, 8.25)
	30–34		3.01.(1.39, 6.54)		2.89 (1.3, 6.4)
	35–39		4.65 (2.31, 9.36)		5.06 (2.45, 10.45)
	40–44		Reference		Reference
	Above 45		0.23 (0.69, 0.77)		0.19 (0.57, 0.65)
Occupational status of the respondent	Farmer		Reference		
	Merchant		1.25 (0.6, 2.6)		
	Government worker		0.46 (0.12, 1.77)		
Sex of the respondent	Male		Reference		
	Female		1.55 (0.94, 2.56)		
Residence	Urban		1.46 (0.93, 2.28)		
	Rural		Reference		Reference
Family awareness about the cause of the disease	Yes		4.17 (2.5, 6.9)		3.6 (2.13, 6.21)
	No		Reference		Reference
Attitude toward SRH	Positive		5.04 (2.73, 9.33)		5.82 (3.16, 10.71)
	Negative		Reference		
Fear of stigma	Yes		1.18 (0.74, 1.89)		
	No		Reference		
Enabling factors					
Occupational status of the partner	Farmer			Reference	
	Merchant			2.35 (1.16, 4.78)	
	Government worker			0.46 (1.28, 1.62)	
Support from Family	Yes			3.25 (2.08, 5.07)	2.47 (1.47, 4.14)
	No			Reference	Ref
Presence of health facility near home	Yes			1.49 (0.87, 2.56)	
	No			Reference	
Involve in income-generating activities	Yes			1.41 (0.92, 2.16)	
	No			Reference	Reference
Able to use household money to health affairs	Yes			1.89 (1.14, 3.12)	2.05 (1.18, 3.57)
	No			Reference	Reference
Wealth index	Poor			Reference	Reference
	Middle			1.98 (1.27, 3.09)	2.32 (1.41, 3.82)
	Rich			2.23 (1.34, 3.7)	3.24 (1.79, 5.88)
Need factors					
Health service use experience	Yes			3.79 (1.99, 7.2)	2.39 (1.56, 4.93)
	No			Reference	Reference

The ICC value declined across subsequent models: 8.4% in Model II (individual-level factors), 1% in Model III (community-level factors), and 6.6% in Model IV (combined individual- and community-level factors). Similarly, the proportional change in variance (PCV) indicated that the predictor variables progressively explained more of the variability observed in the null model. The PCV was 25% for Model II, 26.7% for Model III, and 4.17% for Model IV.

Model IV, which incorporated both individual and community-level predisposing, enabling, and need factors, accounted for 96% of the community-level variance in SRH service utilization. In addition, the AIC and BIC values showed a successive reduction across models, indicating substantial improvement in the model fit at each step. Model IV had the lowest AIC (580.3) and the highest log-likelihood ratio (−275.13), confirming it as the best-fitting model for predicting SRH service utilization among patients with podoconiosis. Consequently, the findings reported are based on Model IV (Table 4).

**Table 4:** Random effect analysis for the assessment of predisposing, enabling, and need factors associated with SRH service use among patients with podoconiosis in Wolaita Zone, South Ethiopia 2024.

Random effect	Null model	Model II	Model III	Model IV
ICC %	11%	8.4%	1%	6.6%
Median odds ratio	2.11	1.91	1.74	1.76
Proportional change in variance (%)	Reference	25.75%	26.7%	4.17%
Model comparison statistics				
Log-likelihood	−394.974	−290.86	−355.1	−275.16
AIC	793.95	607.73	732.6	580.3
BIC	803.4	669.2	784.6	651.24

#### Predisposing, enabling, and need factors (classified as individual and community level) associated with sexual and reproductive health service utilization among patients with podoconiosis

After adjusting for predisposing, enabling, and need factors (can be classified as individual and community-level factors), the odds of SRH utilization were 3. 3.57 (95% CI: 1.56, 8.25) times higher among patients with podoconiosis who were between the ages of 18 and 29 than those who were between the ages of 40 and 44. Those between the ages of 30 and 34 used SRH services 2.89 (95% CI: 1.3–6.4) times more likely than those between the ages of 40 and 44. Similarly, patients with podoconiosis who were 35–39 years old had a 5.06 (95% CI: 2.45–10.45) times higher likelihood of using SRH than those in the age group of 40–44.

According to this study, patients with podoconiosis were 1.6 (95% CI: 1.03–2.5) times more likely than their rural counterparts to use SRH services. Furthermore, the current study found that individuals with podoconiosis whose families knew the etiology of the disease were 3.6 (2.13–6.21) times more likely to use SRH services than patients whose families did not know the cause of the disease. Patients with podoconiosis who had a positive attitude toward SRH services were 5.82 (95% CI: 3.16–10.71) times more likely to use SRH services than those with an unfavorable attitude.

According to this study, patients with podoconiosis who were able to use the money of the household for healthcare were 2.05 (95% CI: 1.183–3.57) times more likely to utilize SRH services than those who were not able to use household money for healthcare.

The current study revealed that respondents in the middle wealth group were 2.32 (95% CI: 1.41, 3.82) times more likely to use SRH services compared with the poor wealth group, and similarly podoconiosis patients from the rich wealth group were 3.24 (95% CI: 1.79, 5.88) times more likely to use SRH services than those from the poor wealth group.

Patients with podoconiosis who had experience of using healthcare services were 2.39 (95% CI: 1.56, 4.93) times more likely to use SRH services than those who had no experience of using healthcare services (Table 3).

## Discussion

The purpose of this study was to identify the magnitude and the multilevel factors associated with the utilization of SRH services among patients with podoconiosis in Wolaita Zone, South Ethiopia. The finding of this study highlighted the multifaceted nature of factors affecting SRH service utilization. This study revealed that SRH utilization among patients with podoconiosis was 18.4% with 95% CI (16%, 21%). The finding is comparable to those of previous studies conducted among other population groups: 19.18% (31) among young girls in rural Ethiopia, 16.89% (32) among adolescents in Southern Ethiopia, and 19.3% (33) among young people in refuge in Uganda. This similarity might be attributed to the fact that these population groups face different challenges to access SRH services due to inaccessibility of health facilities, lack of transportation, healthcare infrastructures that are not inclusive of patients with podoconiosis, poor knowledge of benefits of SRH services among patients, fear of stigmas from community members as well as health professionals, absence of trained health professionals to handle SRH of patients, and absence/poor integration of the services with other podoconiosis treatment services.

The current finding shows lower rates than that of other studies: 23.5% (34) from eastern Ethiopia, 32.8% (35) from southern Ethiopia, 39.5% (36) from Dire Dawa Eastern Ethiopia, 45.5% (37) among Kombolcha industry workers, 54.4% (38) among construction workers in Southern Ethiopia and from other countries, 23.4% from Nigeria (39), and 24.7% from Nepal (40) among adolescents. This discrepancy may be due to the fact that these populations face various obstacles in obtaining SRH services, such as difficulty in accessing medical facilities, lack of transportation, healthcare infrastructures that do not accommodate patients with podoconiosis, lack of awareness among patients about the advantages of SRH services, their fear of stigma from both the community and medical professionals, the lack of trained medical personnel to manage SRH of patients, and the lack or inadequate integration of SRH services with other podoconiosis treatment services.

The current study found a significant association between age and SRH service utilization. Patients aged 18–29 were 3.57 times more likely to utilize SRH services compared with those aged 40–44. Likewise, participants aged 30–34 had 2.89 times higher odds of using SRH services, and those aged 35–39 were 5.06 times more likely to utilize SRH services relative to the 40–44 age group. This finding is consistent with previous research showing that younger persons, particularly those in their reproductive years, are more likely to seek SRH services due to increased knowledge, higher health-seeking habits, and a larger need for family planning or maternal healthcare (41). This similarity might be related to the fact that younger persons are more likely to be exposed to health education programs promoting SRH utilization than older persons, as such programs initiate younger ones to utilize the services.

Furthermore, this finding is comparable to those of studies conducted in Gulliso woreda, West Ethiopia (9), Dembecha district, North Ethiopia (42), and Musanze district, Rwanda (43). This could be due to the fact that younger patients with podoconiosis are more likely to seek SRH services, possibly due to increased health awareness, reproductive health needs, or fewer disease-related mobility limits than older patients.

This study revealed that podoconiosis patients with prior experience in using health services were significantly more likely to utilize SRH services, with odds 2.39 times higher (95% CI: 1.56–4.93) than those without such experience. This finding is similar to that of studies conducted in Baso Liben Woreda, northwestern Ethiopia (44), which showed that 72.8% of patients who participated in a study evaluating their willingness to pay for podoconiosis treatment services said they would be willing to do so, suggesting that people who had previously interacted with the health system valued healthcare services. This finding is also supported by that of another study from South Ethiopia (45), which showed that previous encounters with healthcare providers have a beneficial impact on subsequent health service consumption. This might be attributed to the fact that individuals with podoconiosis are more likely to use SRH services because of their past experiences with healthcare, which encourages a proactive approach to health-seeking behavior, and patients who are acquainted with healthcare facilities are more likely to seek care for a variety of health conditions, including SRH needs.

The study further showed that podoconiosis patients whose families were aware of the etiology of the disease were 3.6 times more likely to utilize SRH services (95% CI: 2.13–6.21) compared with those whose families lacked such knowledge. This finding is comparable with those of studies conducted in South Ethiopia (13) and Rwanda (46), which showed that a lack of knowledge about the illness frequently causes family members of patients to feel more anxious and distressed. Social isolation and stigma brought on by this ignorance may discourage patients from obtaining essential medical care. This could be due to the fact that families that are aware of the etiology of the disease are more equipped to help affected individuals get complete healthcare, including SRH treatments.

Podoconiosis patients with a positive attitude toward SRH services were 5.8 times more likely to utilize these services (95% CI: 3.16–10.7) compared with those with an unfavorable attitude. This finding is consistent with those of other studies conducted in Wolaita Sodo, Kombolcha, and Jimma from Ethiopia, respectively (37, 38, 47, 48).

This study found that patients with podoconiosis who had family support were 2.47 times more likely to utilize SRH services (95% CI: 1.47–4.14) compared with those without family support. This finding is supported by those of other studies from Gamo Zone South Ethiopia (32) among adolescents (49–51). This could be attributed to the fact that supportive family situations can offer emotional support, allow access to information, and reduce the stigma associated with accessing SRH services.

The study also revealed that podoconiosis patients with autonomy to use household money for healthcare were 2.05 times more likely to utilize SRH services (95% CI: 1.18–3.57) compared with those without such autonomy. This result is comparable with studies conducted in Ethiopia (52, 53), Nepal (54), Albania (55), and India (56). This can be due to the fact that having financial independence within the home allows people to prioritize and seek out SRH and other essential healthcare. Giving patients financial control over their homes may enhance health outcomes by enabling prompt and appropriate use of medical services.

## Limitations of the study

Despite the strengths of the study, there are also some limitations that need to be considered while interpreting these findings. First: Self-Report Bias: relying on self-reported data for SRH service utilization may result in recollection bias or social desirability bias, in which participants may over- or under-report their activities: Second: Because this is a cross-sectional study, data are collected at a single point in time, which limits the capacity to prove causality between variables. Third: Since this study is the first to assess SRH utilization among patients with podoconiosis, we used related literature for discussion because of a lack of studies on this specific population. Fourth: Some limitations are related to the use of the theoretical model.

## Conclusion

According to this study, the proportion of patients with podoconiosis who utilized SRH services in the past 12 months was relatively low at 18.4%. Significant factors associated with SRH service utilization included age, prior experience with health services, family awareness of the etiology of the disease, positive attitudes toward SRH services, family support, and autonomy in using household resources for healthcare. Therefore, the following recommendations should be considered to enhance SRH service utilization among patients with podoconiosis.

• District health offices in collaboration with health facilities have to expand community awareness initiatives that highlight the causes of podoconiosis and the value of SRH services for patients with podoconiosis.
• Heath professionals including health extension workers develop family-centered education sessions that highlight how family members can help patients get SRH care.
• Health facilities have to create age-appropriate interventions to improve SRH service use even further, especially for those between the ages of 18 and 39.
• Governmental and non-governmental organizations have to work together to increase the accessibility and availability of SRH services in underserved or rural areas where podoconiosis is common.
• Health facilities would initiate integrating SRH services with routine podoconiosis care.
• Positive views toward SRH services must be encouraged among patients with podoconiosis and their families and their communities, and behavior change communication (BCC) techniques must be used.
• Governmental and non-governmental organizations working on podoconiosis prevention should strengthen family and community support regarding podoconiosis management.
• Health sectors should train and equip health professionals, including health extension workers, for the management of podoconiosis at each health facility.
• Local and national media should strengthen the promotion of podoconiosis management and service availability.
• Local and national policies and strategies should include patients with podoconiosis at the design and intervention levels.
• BCC has to be strengthened by the use of mass media, social media, and local outreach to dispel myths and stigma around SRH services.
• Peer-education programs should be initiated; people who have successfully used SRH programs should be trained to become peer educators, sharing their good experiences to impact community views.

## Data Availability

The original contributions presented in the study are included in the article/Supplementary Material, further inquiries can be directed to the corresponding author.
